# The effect of volunteer-led activities on the quality of life of volunteers, residents, and employees of a long-term care institution: a cohort study

**DOI:** 10.1186/s12877-023-03898-y

**Published:** 2023-03-20

**Authors:** Luisa Veras de Sandes-Guimarães, Patrícia Carla dos Santos, Carla Patricia Grossi Palácio Alves, Carina Junqueira Cervato, Ana Paula Alves Silva, Eliseth Ribeiro Leão

**Affiliations:** 1 Programa de Pós-Graduação em Administração, Universidade Municipal de São Caetano do Sul, São Caetano do Sul, SP Brazil; 2 Ministério da Saúde, Brasília, DF Brazil; 3 Hospital Israelita Albert Einstein, São Paulo, SP Brazil; 4 Universidade de São Paulo, São Paulo, SP Brazil

**Keywords:** Volunteers, Residential facilities, Aged, Quality of life, COVID-19

## Abstract

**Background:**

The COVID-19 pandemic primarily impacted long-term care facilities by restricting visiting and circulation of visitors, affecting the quality of life (QoL) of older adults living in these institutions. Volunteer activities, essential for older adults’ daily life, were also interrupted and potentially negatively impacted the QoL of older adults, volunteers themselves, and also employees in these institutions. In this context, this study aims to evaluate the impact of the return of volunteer-led activities in a long-term care institution on the QoL of older adult residents, employees, and volunteers.

**Methods:**

This study used a pre-test and post-test design within the same group. The first round of data collection was conducted before volunteer-led activities returned and the second round after 1 month of return. The instrument used to assess QoL was the EUROHIS-QoL-8 scale. This study was conducted within a nursing home in São Paulo, Brazil, created in 1937 by members of the Israeli community living in Brazil. Volunteer-led activities were part of residents’ daily life before the COVID-19 pandemic, when these activities were interrupted for about 20 months. A total of 79 individuals participated in both rounds (pre and post), of which: 29 residents, 27 volunteers, and 23 employees of the long-term care institution.

**Results:**

Using a Wilcoxon signed-rank test, the analyses indicated improvements after the 1 month return in different QoL aspects for the three groups. Volunteers improved their personal relationships (Z − 2.332, *p* < .05), residents their overall health (Z − 2.409, *p* < .05) and employees in their overall QoL perception (Z − 2.714, *p* < .05). Influencing factors for residents were the number of activities (3 or more), gender (male), and education (undergraduate/graduate). For employees, those who assumed additional activities due to the volunteer-led activities interruption had a significant impact on their overall QoL.

**Conclusions:**

Evidence from this study suggests that volunteers’ return positively impacted different QoL aspects for volunteers, residents, and employees.

## Background

The COVID-19 pandemic severely affected older adults, one of the most vulnerable groups considering risk factors such as having a lower immunity and the presence of chronic diseases or other comorbidities. Higher mortality rates due to the disease were reported worldwide before the vaccine was made widely available, and older adults living in long-term care facilities (nursing homes or residential care facilities) were even more vulnerable [1]. Several countries imposed restrictions on external visits and internal activities to contain the spread of the disease [2–4], which was indeed more dangerous within institutions where highly susceptible people were clustered in one place [1] and where higher mortality rates due to the disease were reported [5]. The lack of activities and family visits affected the older adults living in these institutions worldwide, who were further isolated due to the demands of the restrictive measures, presenting increased depressive symptoms and anxiety, reduced mental acuteness, physical capacity, well-being, and quality of life due to the lack of social connectedness and other factors [6, 7].

These long-term care institutions and hospitals frequently count on the service of volunteers who perform several non-medical activities associated with the older adults’ daily lives, such as walking, reading, befriending and talking, organizing entertainment and socialization activities, and assisting in nutrition and hydration [2, 4, 8, 9]. Volunteers offer extra assistance and companionship to residents, provide support to the employees (e.g., nurses, nutritionists, and physical therapists), and potentially improve the overall quality of care [8, 10]. The literature presents extensive evidence on the effects of volunteer activity on the volunteers themselves, especially when older adults are concerned (see [11] and [12] for literature reviews on the subject). Such effects include but are not limited to improving life satisfaction, self-esteem, quality of life, physical health, psychological well-being, and reducing depressive symptoms. Some studies also provide evidence for this effect in long-term care facilities or hospital environments, addressing the benefits from an institutional [8, 10, 13] and from the patients’ perspective [8, 14].

Few studies have analyzed the impacts of the volunteer activities interrupted due to the COVID-19 pandemic on healthcare institutions, their patients, and volunteers, and reported increased staff workload [2] and negative impacts on the health and well-being of older adult volunteers [15]. The literature in the context of long-term care institutions (disregarding volunteer activity specifically) has reported negative impacts of the restrictions on residents, which include reduced mental well-being [16], cognitive performance, quality of life [17],functional decline [7], as well as increased feelings of anxiety, of loneliness, and depressive symptoms [18]. Other studies have also reported negative effects on family members and visitors of the residents, reporting low emotional and psychological well-being [19], and feelings of anxiety, frustration, and guilt [18]. To the best of our knowledge, no studies have analyzed this impact in long-term care institutions considering the perspectives of volunteers, residents, and employees of the institutions. Hence, this study contributes to the existing literature by evaluating the impact of the return of volunteer activities in a long-term care institution on the quality of life of older adult residents, employees, and volunteers.

Quality of life (QoL) was selected as the outcome to be evaluated because volunteer-led activities in the long-term care institution analyzed covered physical, emotional, and social aspects of older adults’ daily lives. QoL was never measured at the institution; however, the activities developed by the volunteers may have enhanced residents’ well-being and overall QoL. In addition, volunteering is suggested to lessen possible quality of life reductions, especially considering senior volunteers (+ 50) [12]. QoL is measured based on the individuals’ perceptions (a subjective measure of well-being), focusing on their overall satisfaction or dissatisfaction with different aspects of their lives, e.g., physical and mental/emotional well-being, social relationships, and environment [20]. A limited number of studies have assessed the QoL of older adults living in long-term care facilities during the COVID-19 pandemics [5, 20] using a pre-post intervention design such as in this study.

## Methods

### Study setting

This study was conducted within a nursing home in São Paulo, Brazil, created in 1937 by members of the Israeli community living in Brazil. As of April 2022, the institution currently accommodates 114 elderly people aged from 63 to 105 years old who live in individual rooms and rely on a multidisciplinary team of 269 professionals, including doctors, nurses, physiotherapists, nutritionists, administrators, caregivers, hygiene, nutrition, and maintenance professionals.

The institution also counts on a team of 92 volunteers (April 2022), managed by the volunteer department, who conduct several activities with the residents, contributing to their QoL and well-being. These activities include efforts to improve residents’ QoL in terms of their physical (e.g., craftsmanship, manual work, floral art), emotional/psychological (e.g., memory project, beauty space, Reiki) and social (games and entertainment, library, movie sessions, visiting of volunteers in the residents’ rooms) capacities.

Due to the COVID-19 pandemic, all the activities conducted by volunteers in the institution were entirely interrupted in March 2020 and external visiting became restricted, but all other internal activities remained the same. Volunteers adapted and conducted two activities remotely during this period, namely making phone calls to residents and establishing a video channel with curated content and special remote activities. On-site volunteer-led activities resumed in November 2021 and were interrupted again in mid-January 2022 with the increased number of cases of the omicron variant of the new coronavirus.

### Study design and sample

This study used a pre-test and post-test design within the same group (i.e., without a control group) to assess whether the return of the volunteering activities affected the quality of life of residents, employees, and volunteers of the institution. To this end, an online form was answered by participants before (October 2021) and after the volunteers returned (between mid-December 2021 and mid-January 2022). Details on the questionnaire and data collection are available in the next section.

The study population consisted of 121 elderly residents (admitted into the institution until November 2019), 119 employees (working in the morning/afternoon shifts; hired until November 2019), and 65 volunteers (a subset of the total number of volunteers who were able to return to the activities on November 2021).

Residents were included based on their Mini-Mental State Exam (MMSE) score [21]. The standard cut-off point recommended in the literature is 24 points, but studies carried out in Brazil have shown the relevance of considering the education level to determine the MMSE cut-off point [22, 23]. Therefore, we adopted the following cut-off points for this study: illiterate - 21; lower education - 22; middle education - 23; higher education - 24. Based on the MMSE criteria, 30 residents were eligible, and all were recruited for the study. The totality of eligible residents voluntarily agreed to participate and their families were not directly informed about the study.

The final sample of older adults included 30 people for the data collected before volunteers returned (first round) and 29 after the volunteers returned (second round; one of the residents had died). Convenience sampling was used for employees and volunteers, with an online form sent via e-mail to all suitable participants with follow-up e-mails every week for 3 weeks in each sampling round. The final volunteer sample included 53 (first round) and 27 individuals (second round), and the employee sample included 68 (first round) and 23 individuals (second round).

### Data collection and instruments

This study used the EUROHIS-QoL-8 scale to measure the quality of life (QoL). This scale is derived from the WHOQoL-BREF developed by the World Health Organization (WHO) and contains eight items to measure QoL: overall QoL, general health, energy, daily life activities, self-esteem, relationships, finances, and home. All items are positively phrased and measured using a 5-point scale [24]. The validity and reliability of the Brazilian version of the scale have been tested, confirming good psychometric properties to measure QoL in Brazilian populations [25, 26], with measured reliability (Cronbach’s alpha) of 0.72 and 0.81, respectively. For this study, the scale presented a Cronbach’s alpha of 0.837 (before) and 0.867 (after) and a McDonald’s omega of 0.862 (before) and 0.875 (after).

In addition to the EUROHIS-QoL-8 scale items, the questionnaire also contained questions concerning demography (age, gender, education), volunteer-led activities in which residents participated (before and after), the number of hours volunteers worked per week after the return (limited to 8 by the institution), whether the employee assumed additional activities due to volunteers absence and open questions to express how the volunteer-led activities impacted their overall well-being and QoL. A pre-test of the questionnaire was administered to 6 participants (2 from each group) to verify if items were correctly understood. Only a few modifications to the scale explanation were necessary to facilitate understanding.

Data was collected between October 1st and October 31st, 2021 (first round, before volunteers returned) and between December 12th and January 15th (second round, approximately 1 month after the volunteers returned). The RedCap online tool was used for data collection because it is a robust and secure online survey tool that complies with Health Insurance Portability and Accountability Act (HIPAA) standards. For residents, three of the researchers performed the data collection in person to ensure a correct understanding of the questions and avoid problems with the usage of online tools. The online form was sent via e-mail to volunteers and employees by the volunteer department and institution management, respectively, and follow-up e-mails were sent every week for 3 weeks.

### Data analysis

The data were analyzed using the SPSS 24 software (Statistical Package for Social Sciences Inc., IL, USA). Descriptive statistics (number, percentage, mean, and standard deviation) were calculated for the numerical and categorical data. The outcome data (QoL indicators) were tested for normality using the Shapiro-Wilk and Kolmogorov-Smirnov tests, but the results indicated a non-normal distribution. Hence, the differences among the pre-post QoL indicators were analyzed using the Wilcoxon signed-rank nonparametric test. The Wilcoxon test analyzes scores in the two moments (before and after volunteer return), ranks the differences among scores and assigns a sign to this difference (positive, negative, or no difference). The percentage of cases allocated as positive, negative, or with no difference are presented below to describe those who presented increased or reduced scores for each QoL indicator in the scale. The significance level considered was *p* < 0.05. Open-ended responses were analyzed by coding them thematically using categories derived from the participants’ answers. The categories relate to how the return of volunteer-led activities affected each group (volunteers, employees, and residents).

## Results

### Participant characteristics

A total of 79 respondents were included in this study and were distributed among three groups: 27 volunteers (41.5% of the target population), 29 elderly residents (83% of the target population) and 23 employees (19.3% of the target population). Table 1 presents the participants’ characteristics regarding the demographic variables selected and other specific characteristics of the participants in each group. The mean age of the volunteers was 60.59 (SD = 10.08), 92.6% were women, 85.2% had an undergraduate or graduate degree, and the average hours of volunteering per week was 4.33 (SD = 1.78). The mean age of the residents was 84.62 (SD = 7.99), 65.5% were women, 55.2% had high school education, and the average MMSE test score was 27.03 (SD = 2.23). Residents could participate in 11 activities led by volunteers. Thus, we divided the residents into two groups according to the median number of activities in which they participated: (i) 0 to 2 activities (51.7%); (ii) 3 or more activities (48.3%). Lastly, the mean age of the employees was 38.77 (SD = 7.58), 95.7% were women, and 61.9% had an undergraduate or graduate degree. In the first-round employees were asked if they assumed additional tasks due to volunteers’ absence, and in the final sample (considering both rounds), 36.4% stated that they did.

**Table 1:** Summary of the participants’ characteristics

	**Volunteers**		**Residents**		**Employees**	
	Mean	± SD	Mean	± SD	Mean	± SD
**Age (years)**	60.59	± 10.08	84.62	± 7.99	38.77	± 7.58
**Mini-mental (MMSE) test score**			27.03	± 2.23		
**Average hours of volunteering per week**	4.33	± 1.78				
	**Volunteers**		**Residents**		**Employees**	
	n	%	n	%	n	%
**Gender**						
Male	2	7.4	10	34.5	1	4.3
Female	25	92.6	19	65.5	22	95.7
**Education level**						
Primary school	0	0.0	5	17.2	1	4.8
High/secondary school	4	14.8	16	55.2	7	33.3
Undergraduate/Graduate	23	85.2	8	27.6	13	61.9
**Activities after return**						
0 to 2 activities	–	–	15	51.7	–	–
3 or more activities	–	–	14	48.3	–	–
**Additional activities due to volunteer absence**						
Yes	–	–	–	–	8	36.4
No	–	–	–	–	14	63.6
**Total**	27	100	29	100	23	100

One missing data (for the same record) on employees for education level and additional activities was not included in the table

Table 2 presents the results comparing QoL scores for each scale indicator before and after the return of the volunteers using the Wilcoxon signed-rank test. The analysis of positive and negative ranks indicates whether the participants presented a reduction or increase in the QoL indicators after the volunteers returned to the institution for the period analyzed. For volunteers, indicators presented more positive than negative ranks, except indicators 2 (overall health) and 7 (financial resources). The only indicator that presented a significant difference in the score before and after volunteers’ return was personal relationships (*p* < 0.05), with 30% of volunteers reporting improvements in this aspect after the return. Half of the QoL indicators for the residents presented higher positive than negative ranks, but only overall health (*p* < 0.05) exhibited a significant difference before and after, with 52% of residents reporting improvements after volunteers’ return. Finally, for employees, five indicators presented improvements in their perception; however, only overall QoL had a significant difference before and after (*p* < 0.05).

**Table 2:** EUROHIS-QoL-8 scores analyzes with Wilcoxon signed-rank statistics

		Volunteers				Residents				Employees			
		n	%	Sum of Ranks	Z	n	%	Sum of Ranks	Z	n	%	Sum of Ranks	Z
**1. How would you rate your quality of life?**	Negative Ranks	3	11%	10.50	−1.100	7	24%	76.00	−0.791	0	0%	0.00	−2.714*
	Positive Ranks	5	19%	25.50		12	41%	114.00		8	35%	36.00	
	No difference	19	70%	–		10	34%	–		15	65%	–	
**2. How satisfied are you with your health?**	Negative Ranks	6	22%	30.00	−0.277	3	10%	34.00	−2.409*	2	9%	10.50	−1.069
	Positive Ranks	4	15%	25.00		15	52%	137.00		6	26%	25.50	
	No difference	17	63%	–		11	38%	–		15	65%	–	
**3. Do you have enough energy for everyday life?**	Negative Ranks	5	19%	30.00	−0.775	5	17%	31.00	−0.66	1	4%	3.00	−1.342
	Positive Ranks	7	26%	48.00		7	24%	47.00		4	17%	12.00	
	No difference	15	56%	–		17	59%	–		18	78%	–	
**4. How satisfied are you with your ability to perform your daily living activities?**	Negative Ranks	3	11%	10.50	−1.098	10	34%	88.00	−1.147	4	17%	26.50	−0.106
	Positive Ranks	5	19%	25.50		6	21%	48.00		6	26%	28.50	
	No difference	19	70%	–		13	45%	–		13	57%	–	
**5. How satisfied are you with yourself?**	Negative Ranks	3	11%	9.00	−0.877	5	17%	51.50	−0.911	1	4%	7.00	−1.265
	Positive Ranks	4	15%	19.00		11	38%	84.50		6	26%	21.00	
	No difference	20	74%	–		13	45%	–		16	70%	–	
**6. How satisfied are you with your personal relationships?**	Negative Ranks	1	4%	3.50	−2.332*	8	28%	77.50	−0.051	4	17%	22.00	−0.587
	Positive Ranks	8	30%	41.50		9	31%	75.50		4	17%	14.00	
	No difference	18	67%	–		12	41%	–		15	65%	–	
**7. Have you enough money to meet your needs?**	Negative Ranks	4	15%	14.00	−0.816	12	41%	100.00	−1.767	4	17%	14.00	0.000
	Positive Ranks	2	7%	7.00		4	14%	36.00		3	13%	14.00	
	No difference	21	78%	–		13	45%	–		16	70%	–	
**8. How satisfied are you with the conditions of your living place?**	Negative Ranks	5	19%	27.50	0.000	9	31%	81.00	−0.243	7	30%	37.00	−1.809
	Positive Ranks	5	19%	27.50		8	28%	72.00		2	9%	8.00	
	No difference	17	63%	–		12	41%	–		14	61%	–	
**Total**		**27**	100			**29**	100			**23**	100		

Note 1: one missing data (for the same record) on employees for education level and additional activities was not included in the table

Note 2: *p < .05

The following themes arose for each group when the comments in the open section of the questionnaire were analyzed, reinforcing and amplifying the results mentioned above. Volunteers mostly mentioned three aspects: sense of usefulness (7 mentions), self-realization by helping others (7 mentions), and relationship and sociability with residents and other volunteers (6 mentions). Employees mostly mentioned three aspects: less work overload (6 mentions), return to normal routines (3 mentions) and reduced demand in the sector (2 mentions). Residents’ comments varied, but some highlighted being able to do something outside of their rooms, occupying their time with different activities and meeting people/breaking social isolation. Table 3 presents excerpts from the respondents that represent each category determined while analyzing the open-ended questions.

**Table 3:** Excerpts representing the categories defined based on the open-ended questions

**Volunteers**	**Representative excerpts**
*Usefulness*	“I felt useful again. It’s very nice to give to others.” “I feel useful. I think all this moves my day.” “The face-to-face work makes me feel useful.”
*Self-realization*	“The return was emotional for everyone; there were demonstrations of affection, even watery eyes by emotion. My physical and emotional self-esteem has soared. Helping others helps me a lot.”
*Helping others*	“It was good to see different people in my daily life, to receive the affection of the residents saying that we were greatly missed, to help them in the activities, and to see them happy.”
*Relationships and sociability*	“Seeing beloved people (residents, staff, and other volunteers) does me a lot of good.” “Socializing with residents and other volunteers changes your vibratory pattern.”
**Employees**	**Representative excerpts**
*Reduced overload*	“The return helped the caregivers, leaving them less burdened.” “The return was significant, as it reduced some of the service overload. We don’t always have time to talk to them for a long time, only during assistance. The joy of the elderly is evident.”
*Reduced demand in the sector*	“We noticed a lower demand from the elderly concerning medical care.” “It was excellent. The elderly felt more welcomed and, therefore, the demand in my sector decreased.”
*Return to normal routines*	“It was important because it indirectly showed that routines can return to normal.” “It was important because the daily routines are getting back to normal.” “For example, for hairdressing in the beauty salon, we sometimes removed employees from the scales to do this service.”
**Residents**	**Representative excerpts**
*Activities outside the room*	“Happy to be able to perform an activity outside the bedroom.” “We can leave our rooms and occupy our minds with different activities.”
*Occupying time*	“More joy and stimulus for doing something. A light for us!” “Improved in the sense of having something to do, gradually returning.” “Occupying myself with something. Being without an activity is not good.”
*Meeting people*	“I started interacting more with people and improved my physical well-being.” “Meeting people was important.” “Interaction with people. After the return, we started doing pizza Saturdays to gather and tell stories.”

Table 4 presents the relevant control variables that influence the QoL indicators and a statistically significant difference before and after volunteers returned. The indicators that were influenced by the control variables were overall QoL (employees) and overall health (residents).

**Table 4:** Significant differences before and after with control variables

	Residents							Employees	
	Activities during the period		Gender		Education			Assumed additional activities	
	0 to 2	3 or more	Male	Female	Primary	Secondary	Undergrad/Grad	Yes	No
**How would you rate your quality of life?**	–	–	–	–	–	–	–	0.046*	0.102
**How satisfied are you with your health?**	0.221	0.020*	0.014*	0.161	0.257	0.160	0.046*	–	–

*p < .05

The control variables that were relevant to explain the differences in the positive perceptions of the residents concerning their overall health improvement after the volunteers returned were number of activities, gender, and education. The residents who performed three or more activities, were of the male gender, and had undergraduate or graduate degrees were the ones for which the improvement in overall health perception was significant (Table 4). The relevant variable influencing the overall QoL perception of the employees was their assuming additional activities after volunteer-led activities were interrupted in 2020. The effect on overall QoL was significant only for those who reported “yes”.

## Discussion

This study showed the positive effects of the return of volunteering activities after the COVID-19 restrictions on different QoL aspects for volunteers, residents, and employees of a long-term care institution. After 20 months without volunteer-led activities and with family visiting restrictions, a one-month return brought benefits for volunteers in their personal relationships, residents in their overall health, and employees in their overall QoL perception. Other demographic and context-related factors also influenced this outcome. For instance, residents who participated in three or more volunteer-led activities, were of the male gender, and had undergraduate or graduate education, reported a significant positive impact on their overall health. Employees who reported to have assumed additional activities due to the volunteer-led activities interruption had a significant impact on their overall QoL.

Previous studies have reported benefits of volunteering, especially for older adults, suggesting improved psychological well-being [27], physical health [28], quality of life [29], mental health, and reduced depression symptoms [11]. Other studies have highlighted the importance of volunteering in forming social connections and meaningful social relationships [11, 12, 30]. This aligns with our result of improved personal relationships after the volunteering experience, since this enhanced connection with others assists the formation of new relationships and strengthens previous ones, such as family connections and community ties [30]. Responses to the open-ended questions also emphasized the relevance of socialization and the personal realization of the volunteers in helping others by giving and receiving affection.

Studies suggest several benefits from the perspective of employees of an institution receiving volunteers for both hospitals, reducing costs and improving the overall quality of care [2, 10], and employees, reducing workload and care burden [8, 13]. Our findings also indicate that volunteers helped reduce some of the employees’ workload, increasing the overall QoL for those who assumed additional activities during the period without volunteering activities. Open-ended answers highlighted the importance of volunteers in providing additional assistance, reducing the demand for some employees, and lightening the workload. Although to our knowledge, no studies have reported the effect of volunteering in long-term care facilities on the QoL of employees, one reported that volunteers reduced the emotional and physical burden of the staff [13], which is somewhat aligned with QoL indicators.

The literature reports several benefits from volunteer-led activities for individuals in hospital settings, mostly analyzing older adults as the benefitted individuals. Volunteers provide several activities that stimulate patients’ physical and cognitive capacities and are great companions for listening and talking with patients. This special attention and person-centered care foster patients’ comfort, happiness and well-being [8, 10, 13]. Our findings suggest that volunteer-led activities positively impacted residents’ perceptions of their overall health, specifically concerning those who participated in three or more activities. A systematic review focused on the effect of volunteers’ care on older adults also showed a positive impact of the volunteers on health outcomes (nutrition, falls and delirium) [14]. Our findings are also consistent with the activity theory of aging, which argues that the life satisfaction of older adults is directly related to their level of activity and social interaction [31]. Indeed, occupying time with activities outside the bedroom was one of the themes perceived by the residents as relevant for their daily lives post return.

This study adds evidence supporting the positive outcomes of volunteering activities for the three groups studied (volunteers, residents, employees) and provides two novel contributions. The first novelty was analyzing the contributions of volunteer-led activities to the three groups within a long-term care institution. The second was analyzing the effects of before and after the volunteers returned from the COVID-19 restrictions on the QoL of the three groups. To the best of our knowledge, only one study had analyzed the perspectives of patients, volunteers, and staff of volunteer-led interventions in the context of the Hospital Elder Life Program (HELP) in the Netherlands, also providing evidence of the benefits of this volunteer program for the three groups, but this was reported before the COVID-19 pandemic [8]. However, our study adds that all groups benefited from the return of the volunteer activities in different QoL aspects when compared with the period without these activities.

### Limitations

This study had a some limitations. A significant limitation is how generalizable the study is, given the small sample size and single site of data collection. The response rate to the online survey (for volunteers and employees) was lower than expected in the first but especially in the second round, which may have generated a selection bias, once those who really enjoyed or really disliked volunteer-led activities were more prone to answering. In addition, this study did not include a control group to compare pre-post effects of volunteers’ return, which may limit the results. Although the inclusion of a control group was possible with volunteers (who are outside the institution), it would be very difficult to control and limit the interaction between volunteers, employees, and residents in the study setting, restricting the viability of the control group. Lastly, this study analyzed only a month of volunteer-led activities, which is a short period to expect increased QoL for all indicators. The specific QoL improvements identified for the three groups may be transformed into better overall QoL improvements in a longer period of analysis (considering all indicators in the scale) since longer engagement may render long-term benefits which could only be perceived in time. Hence, for future studies, we suggest including different follow-ups (3 months, 6 months, 1 year) to assess these changes in QoL over time. In addition, future research studies could also include the family perspectives of volunteering, which was not assessed in this study, to obtain a more comprehensive perspective of the stakeholders involved in long-term care facilities.

## Conclusion

Overall, this study found that volunteers’ return (after a long period of interruption given the COVID-19 pandemic) positively impacted different QoL aspects for volunteers, residents, and employees. Residents were most affected considering their overall health perception when participating in 3 or more volunteer-led activities. Employees were most affected in their overall QoL perception when they assumed additional activities due to the prolonged interruption of volunteer-led activities. This study has practical implications for long-term care facilities since it indicates that volunteer care and activities are beneficial for these three groups, which may benefit the institution’s overall quality of care. More research is needed, multicentric and with more participants, to determine the broader applicability and conditions of this effect.

## Data Availability

The dataset used for this study is available from the *Figshare* repository, DOI: 10.6084/m9.figshare.19652898.

## Abbreviations

HIPAA: Health Insurance Portability and Accountability Act
MMSE: Mini-Mental State Exam
QoL: Quality of Life
SD: Standard Deviation
WHO: World Health Organization
